# Improving Patient Communication by Simplifying AI-Generated Dental Radiology Reports With ChatGPT: Comparative Study

**DOI:** 10.2196/73337

**Published:** 2025-06-09

**Authors:** Daniel Stephan, Annika S Bertsch, Sophia Schumacher, Behrus Puladi, Matthias Burwinkel, Bilal Al-Nawas, Peer W Kämmerer, Daniel GE Thiem

**Affiliations:** 1 Department of Oral and Maxillofacial Surgery, Facial Plastic Surgery University Medical Centre Johannes Gutenberg-University Mainz Mainz Germany; 2 Department of Oral and Maxillofacial Surgery University Hospital Rheinisch-Westfälische Technische Hochschule Aachen Aachen Germany

**Keywords:** radiology report, patient-centered care, natural language processing, health literacy, medical accessibility, patient comprehension, clinical communication

## Abstract

**Background:**

Medical reports, particularly radiology findings, are often written for professional communication, making them difficult for patients to understand. This communication barrier can reduce patient engagement and lead to misinterpretation. Artificial intelligence (AI), especially large language models such as ChatGPT, offers new opportunities for simplifying medical documentation to improve patient comprehension.

**Objective:**

We aimed to evaluate whether AI-generated radiology reports simplified by ChatGPT improve patient understanding, readability, and communication quality compared to original AI-generated reports.

**Methods:**

In total, 3 versions of radiology reports were created using ChatGPT: an original AI-generated version (text 1), a patient-friendly, simplified version (text 2), and a further simplified and accessibility-optimized version (text 3). A total of 300 patients (n=100, 33.3% per group), excluding patients with medical education, were randomly assigned to review one text version and complete a standardized questionnaire. Readability was assessed using the Flesch Reading Ease (FRE) score and LIX indices.

**Results:**

Both simplified texts showed significantly higher readability scores (text 1: FRE score=51.1; text 2: FRE score=55.0; and text 3: FRE score=56.4; *P*<.001) and lower LIX scores, indicating enhanced clarity. Text 3 had the shortest sentences, had the fewest long words, and scored best on all patient-rated dimensions. Questionnaire results revealed significantly higher ratings for texts 2 and 3 across clarity (*P*<.001), tone (*P*<.001), structure, and patient engagement. For example, patients rated the ability to understand findings without help highest for text 3 (mean 1.5, SD 0.7) and lowest for text 1 (mean 3.1, SD 1.4). Both simplified texts significantly improved patients’ ability to prepare for clinical conversations and promoted shared decision-making.

**Conclusions:**

AI-generated simplification of radiology reports significantly enhances patient comprehension and engagement. These findings highlight the potential of ChatGPT as a tool to improve patient-centered communication. While promising, future research should focus on ensuring clinical accuracy and exploring applications across diverse patient populations to support equitable and effective integration of AI in health care communication.

## Introduction

### Background

The recording and communication of diagnostic findings, treatment plans, and patient progress are fundamental to high-quality patient care [1]. Structured and standardized medical documentation should support 2 essential objectives in health care: it should enable effective communication among medical professionals while also improving therapy involvement of patients. However, the complexity of medical documentation often poses significant barriers, particularly for patients, due to technical language and specialized terminology limiting comprehension. Clear and comprehensible medical information enables patients to better understand their conditions and actively participate in shared decision-making, highlighting the need for innovative approaches to improve accessibility [2,3].

Despite its critical role in health care, traditional manual documentation presents numerous challenges. Writing detailed medical reports is both time-consuming and error-prone, particularly when multiple versions are required to address different audiences, including patients and health care professionals. Health care providers further cope with extensive documentation requirements, driven by legal, regulatory, and insurance standards, contributing to documentation overload [4]. These demands not only impair clinical workflow efficiency but also demonstrate the importance of solutions balancing accuracy, clarity, and usability in fast-paced medical environments.

Artificial intelligence (AI) has emerged as a transformative tool in medicine, expanding its applications beyond disease diagnosis and pharmaceutical drug development [5]. AI technologies have demonstrated remarkable potential across various medical disciplines, particularly in radiology, where advanced algorithms facilitate medical image interpretation. These innovations enable early disease detection, improve diagnostic precision, and support clinical decision-making [6,7]. Furthermore, AI-driven methodologies are increasingly used to optimize and streamline health care workflows, including documentation practices. Due to automating repetitive tasks and translating complex medical nomenclature into comprehensible language, AI addresses persistent inefficiencies [8]. A key advantage is the potential to generate clear, patient-friendly documents without increasing clinicians’ workload. This capability is particularly crucial in radiology, as reports are highly technical and predominantly intended for medical professionals.

Patients are often provided with their radiology reports digitally or as a printed document, frequently without sufficient explanation. This lack of information can result in misinterpretation and confusion. Unfamiliar terms such as “lesion” or “opacity” may cause patients to overestimate the severity of findings, leading to unnecessary anxiety or, conversely, overlooked critical recommendations resulting in delayed or missed follow-ups. In addition, the absence of clear communication in radiology reports may compromise informed consent, leaving patients unable to fully understand the implications of their findings or treatment options. The AI-driven translation of technical medical language into easily comprehensible terms offers the potential to bridge the gap between complex medical findings and patient understanding.

Although traditional methods of simplifying medical language often impose a significantly increased workload, advancements in AI offer a more efficient and comprehensive opportunity. Large language models (LLMs), such as OpenAI’s GPT, which was first introduced in 2018 [9], have gained substantial attention in recent years as transformative tools in natural language processing. GPT has been progressively refined through extensive training, improving its ability to generate and interpret medical texts [10,11]. Notably, ChatGPT, an implementation of GPT, excels in generating coherent, humanlike text by leveraging advanced deep learning techniques, including neural networks, to process and produce language with remarkable precision. ChatGPT’s potential in medical applications is underscored by its strong performance in standardized examinations [12,13], demonstrating its ability to process complex medical content at a level comparable to human expertise. This capability has imparted its integration into clinical practice and is used to automate routine documentation tasks, including drafting examination findings, composing operative notes, and generating radiology reports [14,15].

However, potential limitations regarding the use of AI must be considered. LLMs are known to occasionally generate inaccurate or fabricated content, which is often referred to as hallucinations [16,17]. These outputs may appear linguistically fluent while conveying clinically incorrect information and therefore posing significant risks in medical contexts [10]. In addition, AI-generated content can reflect and even amplify the biases embedded in its training data, potentially resulting in inequities in communication and care recommendations, especially for underrepresented populations. Previous studies have demonstrated that these models can exhibit prejudiced behavior toward speakers of African American English, associating them with negative attributes and thereby perpetuating the racial and cultural biases present in their training data [18,19].

Furthermore, the reliability of AI models in accurately reflecting clinical context across diverse medical scenarios remains uncertain. Although LLMs can generate text that mimics expert-level communication, they lack genuine understanding of medical ethics, individual patient situations, and the complexity of clinical decision-making. Most models are trained primarily on English-language and Western data, which may reduce the effectiveness of AI-generated texts for nonnative speakers due to linguistic or cultural biases, as subtle differences in tone, phrasing, and meaning can be lost or misrepresented [20].

Beyond its utility for health care professionals, ChatGPT nevertheless presents an opportunity to address challenges in patient communication by simplifying complex medical information. This potential to enhance patient communication by generating accessible and comprehensible radiology reports represents an emerging area of interest [8].

While advancements in AI have demonstrated its utility in generating technically accurate and readable medical texts, research on its impact on patient communication remains limited. Most existing studies emphasize the evaluation of technical attributes of AI-generated texts, such as accuracy, grammar, and readability, without adequately addressing their effects on patient understanding or engagement [21].

### Objectives

Therefore, building on previous research, we aimed to investigate the potential of ChatGPT in generating and simplifying radiology reports by primarily analyzing patient evaluations regarding text comprehensibility, clarity, and communication quality of both AI-generated and AI-simplified radiology reports with the null hypothesis stating no differences among the 3 sets of reports. Secondary outcomes, including text quality and readability, were assessed to identify opportunities for improvement in AI-driven radiology reporting.

## Methods

### Overview

This study aimed to evaluate the efficacy of using AI language models, specifically ChatGPT, in simplifying and enhancing the readability of radiology reports to improve patient communication. All radiology reports were fully AI generated. First, checkbox lists of diagnoses were used to generate radiology reports using ChatGPT. Second, ChatGPT was used to create one patient-friendly simplified and a second accessibility-optimized version of these AI-generated reports to assess the impact of simplification on readability and comprehension. The primary evaluation focused on patient responses collected through a questionnaire, while secondary parameters included text quality and readability differences between original AI-generated and simplified AI-generated reports.

### Text Generation

The analysis of dental panoramic radiographs and the generation of radiology reports were not part of this study. Instead, the initial AI-generated reports (based on checkbox lists) were obtained from a previous study [21]. The report generation process, previously described in detail by our group [21], is summarized as follows: panoramic radiographs were analyzed, and structured checkbox lists of diagnoses were completed. These checkbox lists were transcribed into a Microsoft Excel datasheet, with individual spreadsheets created for each diagnostic category to ensure systematic data organization. Subsequently, radiology reports were generated using ChatGPT with GPT-4 (OpenAI), a state-of-the-art AI language model, with the following settings: a temperature of 0.7, a maximum token limit of 1500, a frequency penalty of 0.0, and a presence penalty of 0.6. Each report was generated in a new session of the ChatGPT web interface to maintain consistency and standardization throughout the process using the following prompt:

Formulate a structured X-ray report in the sense of an X-ray report of an OPG [orthopantomagram] based on the following checkbox list of the entire Excel table, and do not omit any columns. Please mention only those statements for which a box is marked with an X in the X-ray report. The statements not marked with an X should not be included in the report. The figures given should be interpreted in the sense of an odontogram. Analyze each spreadsheet in the Excel file in the order given. The column with the markings (X) is marked with the term “checkbox.” The report should be written in continuous text from the perspective of the treating dentist. Formulate a continuous text without subheadings.

### Simplification of Radiology Reports

The AI-generated reports have been analyzed in detail in our previous study [21]. On the basis of these reports, 2 additional simplified versions were created for each AI-generated report. Each of the 100 original AI-generated texts was reformulated into 2 simplified versions using ChatGPT with GPT-4 under the following settings: a temperature of 0.7 (controlling the randomness of responses), a maximum token limit of 1500 (restricting output length), a frequency penalty of 0.0 (preventing repetitive word use), and a presence penalty of 0.6 (encouraging the inclusion of new content), resulting in a total of 300 texts. Reports were generated via the ChatGPT web interface in individual sessions conducted between April 6, 2024, and May 8, 2024. Potential variability in performance due to server load fluctuations and biases in output quality was reduced by random distribution across different weekdays, hence ensuring consistency and reliability.

The following prompts were used to create simplified versions of the reports:

Rewrite the radiology report to make it easier for a patient to understand. Do not leave out any information or content.

Rewrite the radiology report to make it understandable for patients of all educational backgrounds. Do not leave any information or content.

The resulting texts were analyzed for readability and text quality. To minimize bias in the patient evaluation phase, a single text was randomly selected from each group (original AI generated, first simplified, and second simplified). The selected texts were assessed for readability (Flesch Reading Ease [FRE] score and LIX readability index) to ensure that they represent the mean of their respective groups. This approach avoided the inclusion of outliers. These 3 representative texts were subsequently used for patient evaluation.

### Study Setting and Participants

The study was conducted in the Department of Oral and Maxillofacial Surgery, Facial Plastic Surgery at the University Medical Centre Mainz (Germany). Patients were randomly selected and invited to participate. Those who agreed to participate were provided with one randomly assigned text and the corresponding questionnaire. Patients with previous medical education or knowledge, as well as those aged <18 years, were excluded from the study to ensure the data reflected the perspectives of a general audience. No additional exclusion criteria were applied, as the aim was to include a broad and representative sample of the general patient population typically encountered in clinical settings. Both patients and the observer were blinded to the group allocation. The different text versions were anonymized, shuffled, and distributed in a randomized order, so neither the participants nor the observer knew which version was being evaluated. All data were collected anonymously between June 1, 2024, and August 30, 2024, with no personal information recorded. Participants were informed of their right to withdraw consent at any time without consequences. A total of 300 patients participated in the study, with 100 (33.3%) patients assigned to evaluate each text version.

### Text Analysis

To compare the different AI-generated radiology reports, descriptive text analysis was performed. Metrics included word count, sentence count, and proportion of long words (defined as words with more than 6 characters). Because AI-generated texts are inherently free of spelling and grammatical errors, no evaluation of errors or error rates was carried out.

### Readability Indices

The readability and complexity of the texts were assessed using the FRE score [22] and the LIX readability index, as described in detail in our previous publication [21]. These metrics evaluate text comprehensibility based on linguistic features such as sentence length and word complexity.

The FRE score calculates readability based on the average sentence length (*ASL*) and the average number of syllables per word (*ASW*) [23] using the following formula:

*FRE* score = 180 – *ASL* – 58.5 × *ASW*

As the established metric, higher FRE scores indicate more readable texts, while lower scores reflect greater complexity [24,25]. The LIX-Index considers the ASL and the prevalence of long words with more than 6 letters to assess text readability using the following formula:

LIX score = (Total number of words/Total number of sentences) + ([Number of long words × 100]/Total number of words)

Higher LIX scores denote increased complexity, while lower scores suggest simpler texts [26,27]. Both indices are well-established tools validated in medical and nonmedical contexts and provide a robust measure for comparing text readability.

### Experimental Design

Patients were randomly assigned to 1 of the 3 groups, each receiving one text (text 1, text 2, or text 3) along with the corresponding questionnaire. After completing the questionnaire, the responses were collected and analyzed. There were no additional restrictions or limitations imposed in this study, and participants were not subjected to a time limit for completing the questionnaire.

### Questionnaire

The questionnaire consisted of 11 questions divided into 5 thematic categories, as described in Textbox 1.

Questions were answered on a 5-point Likert scale ranging from 1 (“do not agree at all”) to 5 (“fully agree”), with intermediate options: “rather do not agree” (rating=2), “neither agree nor disagree” (rating=3), and “rather agree” (rating=4).

**Thematic categories and questions.**

**Clarity of the report**

- I found the radiology report easy to understand. (Question 1)
- The medical terms in the radiology report were clearly explained. (Question 2)
- I was able to understand the findings of the radiology report without additional help. (Question 3)

**Structure and information content**

- The structure of the radiology report helped me to easily understand the information. (Question 4)
- The information in the radiology report was detailed enough to answer my questions. (Question 5)

**Empathy and tone**

- The tone of the radiology report was respectful and took my situation as a patient into account. (Question 6)
- The radiology report conveyed an empathetic attitude toward my level of understanding. (Question 7)

**Guidance and motivation for action**

- The radiology report helps me ask the right questions to my physician or dentist. (Question 8)
- After reading the radiology report, I can discuss my diagnosis with my physician or dentist and have a discussion on equal terms. (Question 9)
- The radiology report motivates me to focus more on oral hygiene and health in the future. (Question 10)

**Future perspective**

- I would like all my medical reports to be as understandable as this radiology report. (Question 11)

### Ethical Considerations

This study was conducted in strict adherence to ethical guidelines, with informed consent obtained from all participants verbally before their involvement. Confidentiality and data privacy were rigorously upheld throughout the research process to ensure the participants’ well-being and privacy. The local ethics committee (Ethics Committee of the State Medical Association of Rhineland-Palatinate, Mainz, Germany) confirmed that an ethics approval was not required as the study primarily constituted a quality assurance measure, and the survey was conducted anonymously (application 2024-17526). Furthermore, all data were analyzed anonymously. Participants did not receive any form of compensation for their involvement in the study.

### Power Analysis and Sample Size

The sample size for this study was determined through an a priori power analysis based on the primary outcome: patient responses to the questionnaire. A recent study evaluating a patient information leaflet for patients with liver cirrhosis demonstrated a significant improvement in pre- and posttest knowledge scores [28]. Specifically, the study reported an improvement of 4 points (before the test: median 12, range 6-20; after the test: median 16, range 12-22; *P*<.05), corresponding to a moderate effect size (Cohen *d*=0.4). Assuming a similar effect size, to achieve a power of 80% and maintain a significance level of 5%, a minimum of 53 samples per group (159 participants in total) is required. To ensure robustness and account for potential dropouts or incomplete data, the study included 100 participants per group, resulting in a total of 300 participants.

### Statistical Analysis

Statistical analyses were conducted using the following software packages: GraphPad Prism (version 9.0; GraphPad Software, LLC); G*Power (version 3.1; Heinrich Heine University); Excel (version 16.76; Microsoft Corporation); and SPSS Statistics (version 29; IBM Corp). ChatGPT with GPT-4 was used for proofreading. Differences in text quality and readability between different versions of AI-generated texts were analyzed using a 1-way repeated measures ANOVA. Post hoc comparisons were performed with the Tukey multiple comparisons test. All numerical data are presented as mean (SD), with statistical significance set at *P*<.05. Analysis of the patient questionnaire was conducted using an ordinary 1-way ANOVA with the Tukey multiple comparisons test for post hoc comparison. Each graph corresponds to a specific question displayed above the respective graphs, and data are presented as box-and-whisker plots, illustrating medians and IQRs.

## Results

### Overview

#### Background

Text quality, readability, and comprehensibility of AI-generated and AI-simplified radiology reports were evaluated. In addition, patient feedback was integrated to assess the effectiveness of simplified reports in enhancing understanding and engagement with the medical information. The findings demonstrated that simplified versions significantly improved readability and accessibility, aligning with the primary objective of enhancing patient communication. For clarity, the 3 versions of the texts are referred to as follows: the original AI-generated version (text 1), the patient-friendly simplified version (text 2), and the accessibility-optimized version (text 3).

#### Linguistic Characteristics of Simplified Radiology Reports

**Figure 1:**
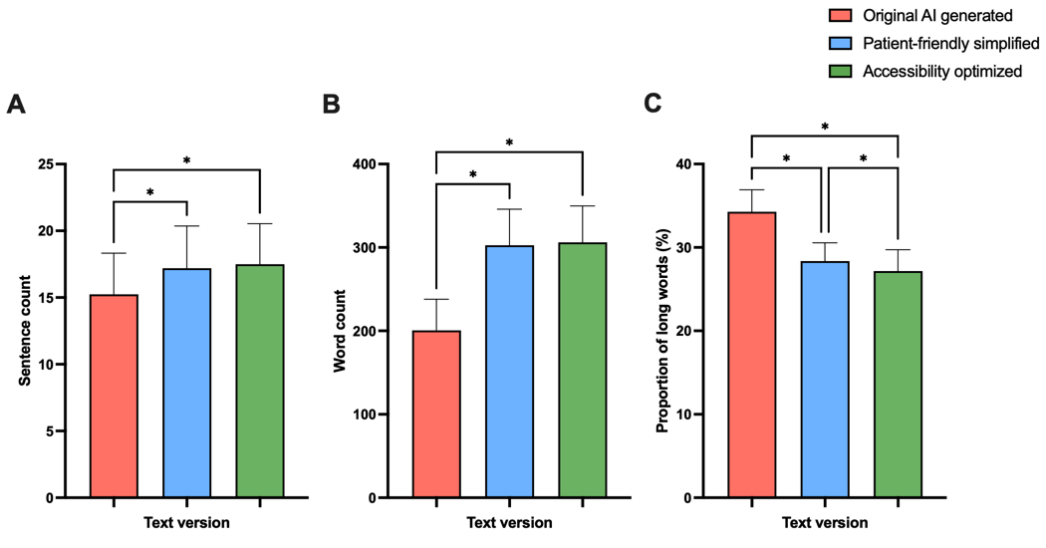
Analysis of (A) sentence count, (B) word count, and (C) proportion of long words in artificial intelligence (AI)–simplified radiology reports compared to original AI-generated reports (n=100). Data represent mean (SD). *P<.05.

The 1-way repeated measures ANOVA revealed a significant difference in sentence count among the 3 text groups, including the AI-generated text and its simplified versions (text 1: mean 15.2, SD 3.1 vs text 2: mean 17.2, SD 3.2 vs text 3: mean 17.5, SD 3.0; *P*<.001; *F*_1.835,181.6_=29.89). In particular, both simplified texts contained fewer sentences as presented in Figure 1, while showing a higher sentence count compared to the original AI-generated text. A similar reduction could be observed in word count, with significant differences across groups (text 1: mean 200.6, SD 37.3 vs text 2: mean 302.6, SD 43.3 vs text 3: mean 306.1, SD 43.8; *P*<.001; *F*_1.72,173.3_=424.9). The original AI-generated report contained significantly fewer words than both simplified versions (texts 2 and 3), with no significant difference in word count between texts 2 and 3. These differences were also reflected in the proportion of long words, which showed a significant difference across all groups (*P*<.001; *F*_1.924,190.5_=294.5) as presented in Figure 1. Post hoc analysis revealed a stepwise reduction in the proportion of long words, with a substantial decrease from the original AI-generated texts to the patient-friendly simplified version (text 2), and a further reduction observed in text 3, the accessibility-optimized version.

#### Improved Readability With Simplified Text Versions

**Figure 2:**
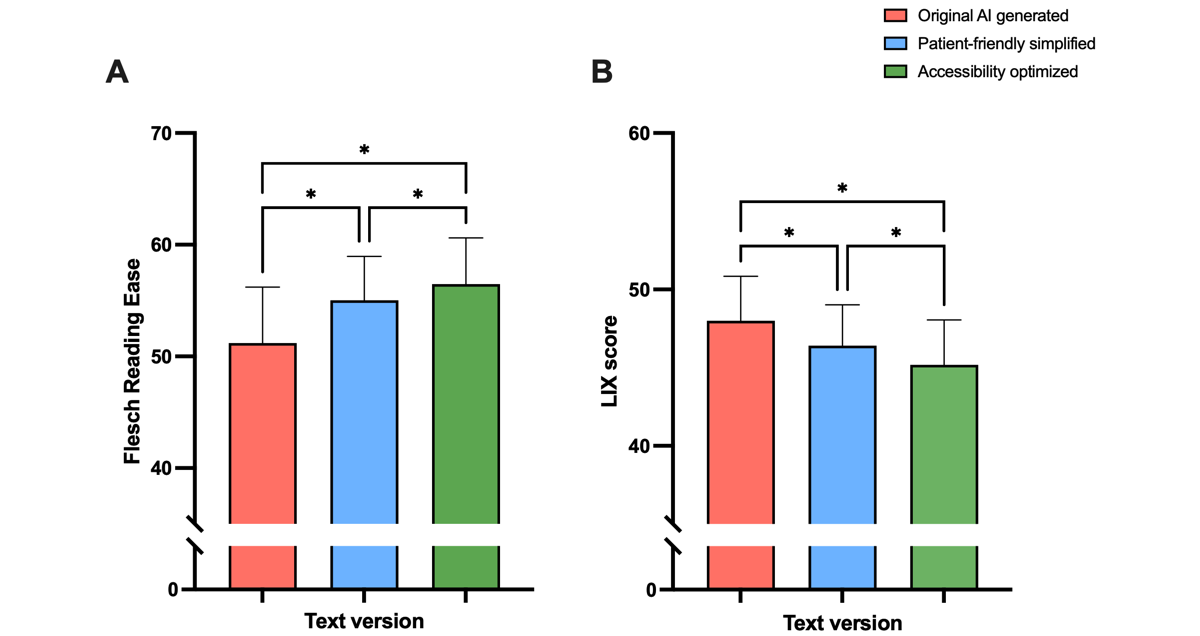
Metric evaluation of readability of artificial intelligence (AI)–simplified reports and original AI-generated reports assessed with (A) the Flesch Reading Ease score and (B) the LIX score (n=100). Data represent mean (SD). *P<.05.

Significant differences in readability were observed across the 3 text groups, as identified by the 1-way repeated measures ANOVA (FRE score: *P*<.001; *F*_1.997,197.7_=47.26; Figure 2A and LIX score: *P*<.001; *F*_1.997,195.7_=31.02; Figure 2B). Text 1 showed significantly lower FRE scores compared to the simplified texts (texts 2 and 3), indicating improved readability due to AI simplification. Text 3 displayed a significantly higher score than text 2. Similar results could be observed in the LIX scores as depicted in Figure 2B. LIX scores for text 1 were significantly higher than those of texts 2 and 3, indicating greater complexity, with text 3 being the simplest and most readable text compared to the other groups.

#### Descriptive Statistics of Questionnaire Responses: Comparison of Patients’ Perceptions of AI-Generated and Simplified Texts

Table 1 provides a summary of descriptive statistics for patient responses to text 1 (AI-generated radiology reports) across 11 survey questions. Median ratings ranged from 2 to 4, with question 2 receiving the highest score (mean 3.2, SD 1.3) and question 6 the lowest (mean 2.1, SD 1.1). The overall range of responses spanned from 1 (minimum) to 5 (maximum), reflecting variability in patient perceptions of clarity, tone, and informativeness. Coefficients of variation were generally moderate, indicating consistent responses across most questions.

**Table 1:** Descriptive statistics for questionnaire responses to the original artificial intelligence–generated radiology reports.

Question number	Sample size (n=100), n (%)	Ratings, median (range; IQR)	Ratings, mean (SD: 95% CI)	Ratings, SEM	Ratings, COE^a^ (%)
1	99 (99)	4 (2-4; 3-5)	2.7 (1.2; 2.47-2.93)	0.12	42.85
2	100 (100)	4 (2-4; 4-5)	3.2 (1.3; 2.90-3.44)	0.13	42.31
3	100 (100)	4 (2-4; 3-5)	3.1 (1.4; 2.78-3.32)	0.14	44.55
4	100 (100)	4 (2-4; 3-5)	2.9 (1.3; 2.60-3.10)	0.13	44.15
5	100 (100)	4 (2-4; 3-5)	2.9 (1.3; 2.60-3.10)	0.13	44.43
6	100 (100)	3 (1-4; 2-5)	2.1 (1.1; 1.89-2.33)	0.11	51.70
7	99 (99)	4 (2-4; 3-5)	2.8 (1.1; 2.63-3.05)	0.11	37.57
8	98 (98)	4 (2-4; 2-5)	2.1 (1.0; 1.90-2.31)	0.10	48.54
9	100 (100)	4 (2-4; 2.5-5)	2.8 (1.3; 2.51-3.03)	0.13	46.99
10	100 (100)	4 (2-4; 3-5)	2.7 (1.3; 2.43-2.95)	0.13	48.91
11	100 (100)	4 (2-4; 3-5)	2.7 (1.4; 2.44-2.99)	0.14	51.20

^a^COE: coefficient of variation.

Table 2 summarizes descriptive statistics for patient responses to text 2 (patient-friendly simplified AI-generated radiology reports) for all 11 survey questions. Median ratings generally ranged from 1 to 2, with lower mean scores compared to text 1, such as mean 1.6 (SD 0.8) for question 1 and mean 1.7 (SD 1.0) for question 11. The overall response range remained consistent from 1 to 5, indicating variability in patient perceptions. SDs were slightly lower than those of text 1, suggesting more consistent responses. The coefficients of variation highlight moderate variability across questions, with question 7 showing one of the highest at 57.04%.

**Table 2:** Descriptive statistics for questionnaire responses to the patient-friendly simplified radiology reports.

Question number	Sample size (n=100), n (%)	Ratings, median (range; IQR)	Ratings, mean (SD; 95% CI)	Ratings, SEM	Ratings, COE^a^ (%)
1	100 (100)	1 (1-5; 1-2)	1.6 (0.8; 1.41-1.74)	0.08	52.99
2	100 (100)	1 (1-4; 1-2)	1.6 (0.7; 1.41-1.69)	0.07	46.19
3	100 (100)	1 (1-5; 1-2)	1.6 (0.9; 1.42-1.80)	0.09	58.49
4	100 (100)	1 (1-5; 1-2)	1.6 (1.0; 1.42-1.80)	0.10	60.46
5	99 (99)	1 (1-5; 1-2)	1.7 (0.9; 1.48-1.85)	0.09	55.55
6	100 (100)	1 (1-4; 1-2)	1.4 (0.7; 1.30-1.58)	0.07	48.68
7	99 (99)	1 (1-5; 1-2)	1.7 (1.0; 1.49-1.87)	0.10	57.04
8	100 (100)	1 (1-5; 1-2)	1.5 (0.8; 1.3-1.6)	0.08	54.02
9	100 (100)	2 (1-5; 1-2)	1.7 (0.9; 1.52-1.86)	0.09	50.95
10	100 (100)	2 (1-5; 1-3)	2.1 (1.1; 1.92-2.36)	0.11	51.03
11	100 (100)	1 (1-5; 1-2)	1.7 (1.0; 1.45-1.85)	0.10	61.14

^a^COE: coefficient of variation.

Table 3 provides an overview of descriptive statistics for patient responses to text 3 (accessibility-optimized AI-generated radiology reports). Median ratings were consistently lower, ranging from 1 to 2, with mean scores slightly reduced compared to text 2 (eg, question 1: mean 1.5, SD 0.7; question 11: mean 1.6, SD 0.8). The response range remained between 1 and 5, showing variability in patient perceptions. SDs and coefficients of variation were generally similar to text 2, with the highest variability observed in question 7 (59.20%).

**Table 3:** Descriptive statistics for questionnaire responses to the accessibility-optimized radiology reports.

Question number	Sample size (n=100), n (%)	Ratings, median (range; IQR)	Ratings, mean (SD; 95% CI)	Ratings, SEM	Ratings, COE^a^ (%)
1	100 (100)	1 (1-4; 1-2)	1.5 (0.7; 1.38-1.67)	0.07	48.12
2	100 (100)	1 (1-4; 1-2)	1.6 (0.8; 1.43-1.77)	0.08	52.55
3	100 (100)	1 (1-4; 1-2)	1.5 (0.7; 1.33-1.61)	0.07	48.78
4	100 (100)	1 (1-5; 1-2)	1.7 (0.9; 1.50-1.86)	0.09	53.43
5	100 (100)	2 (1-5; 1-2)	1.9 (1.1; 1.71-2.15)	0.11	56.69
6	100 (100)	1 (1-5; 1-2)	1.5 (0.8; 1.33-1.65)	0.08	55.21
7	1000	2 (1-5; 1-2)	1.7 (0.8; 1.53-1.85)	0.08	46.60
8	100 (100)	1 (1-5; 1-2)	1.9 (1.1; 1.65-2.09)	0.11	59.20
9	100 (100)	2 (1-5; 1-2)	1.9 (1.0; 1.72-2.10)	0.10	50.52
10	100 (100)	2 (1-5; 1-3)	2.1 (1.0; 1.87-2.27)	0.10	47.70
11	100 (100)	1 (1-5; 1-2)	1.6 (0.8; 1.40-1.73)	0.08	53.38

^a^COE: coefficient of variation.

### Patient Survey Responses Regarding the Comprehensibility of Radiology Reports

#### Overview

Text clarity, structure and information content, empathy and tone, motivation for action, and future perspective of AI-generated radiology reports and their AI-simplified versions were evaluated through patient survey responses. In total, 3 text groups were assessed: text 1 (original AI generated), text 2 (patient-friendly, simplified AI generated), and text 3 (accessibility-optimized AI generated). Each participant completed a questionnaire referring to only 1 text version, and multiple participations were not allowed. Patients with previous medical knowledge or education were excluded from the survey to ensure responses reflected the perceptions of a general audience.

Questions were answered on a 5-point Likert scale ranging from 1 (“do not agree at all”) to 5 (“fully agree”), with intermediate options: “rather do not agree,” “neither agree nor disagree,” and “rather agree.” Each graph corresponds to a specific question displayed above the respective graphs, with data being presented as box-and-whisker plots, showing medians and IQRs. Both simplified report versions (texts 2 and 3) received significantly better ratings across all evaluated aspects, highlighting a preference for more accessible medical documents in the future.

#### Clarity of the Report

**Figure 3:**
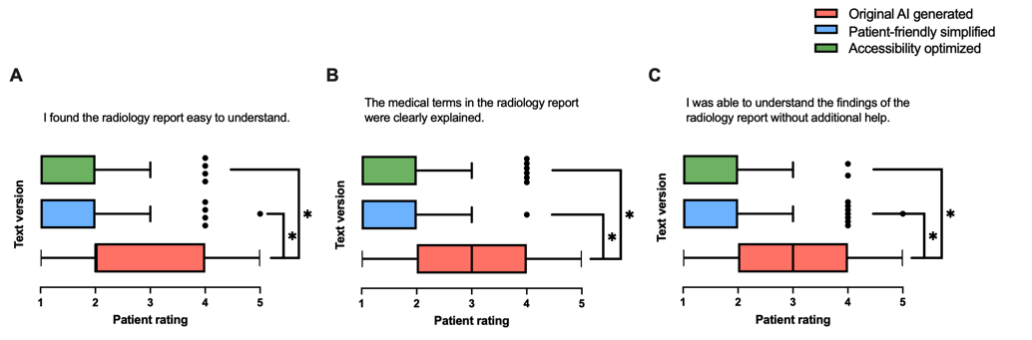
Patient ratings of their comprehension of radiology reports for original artificial intelligence (AI)–generated (text 1), patient-friendly, simplified AI-generated (text 2), and accessibility-optimized AI-generated (text 3) reports. Responses were assessed for (A) ease of understanding the radiology reports, (B) clarity of the medical terms, and (C) ability to understand the findings without additional help (n=100). Data are presented as box-and-whisker plots with medians and IQRs. *P<.05.

As presented in Figure 3, significant differences were identified among the 3 text versions. The simplified text versions significantly improved patients’ ability to understand the information (Figure 3A; *F*_2,296_=51.61; *P*<.001); the clarity of medical terms (Figure 3B; *F*_2,297_=84.34; *P*<.001); and the ability to comprehend the findings of the report without additional help (Figure 3C; *F*_2,297_=70.68; *P*<.001). Throughout all questions regarding clarity and comprehensibility, text 3 received the highest ratings, followed by text 2, with both being significantly higher than text 1.

#### Structure and Information Content

**Figure 4:**
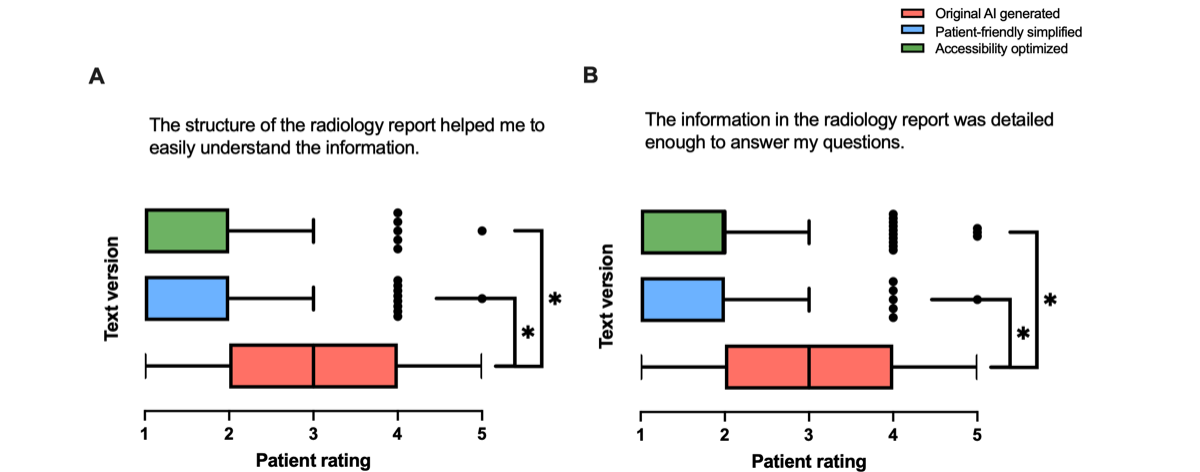
Patient ratings comparing original artificial intelligence (AI)–generated (text 1), patient-friendly simplified AI-generated (text 2), and accessibility-optimized AI-generated (text 3) radiology reports. Ratings were assessed for (A) how the structure of the report helped in understanding the information and (B) whether the information in the report was detailed enough to answer patients’ questions (n=100). Data are presented as box-and-whisker plots, showing medians and IQRs. *P<.05.

The 3 text versions demonstrated significant differences in how patients perceived the structure and information content of the radiology reports. Patients rated the structure of the report as being most helpful for understanding the information in the simplified versions (Figure 4A; *F*_2,297_=43.63; *P*<.001). Similarly, the simplified texts were evaluated as containing sufficient detail to adequately answer patient questions (Figure 4B; *F*_2,296_=31.53; *P*<.001). Text 3 demonstrated the highest ratings across both measures, followed by text 2, with text 1 showing lower medians. All those results are significant.

#### Empathy and Tone

**Figure 5:**
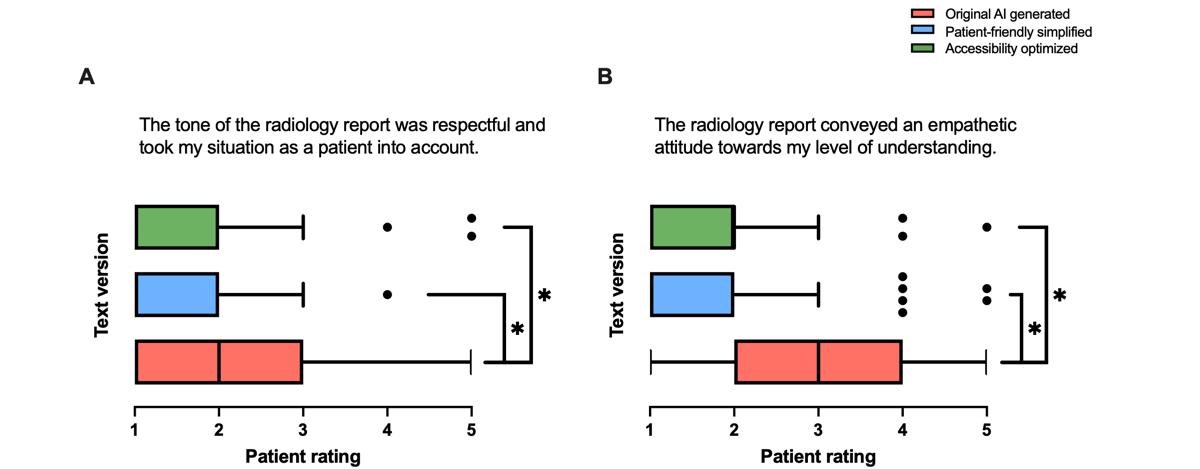
Patient ratings comparing original artificial intelligence (AI)–generated (text 1), patient-friendly simplified AI-generated (text 2), and accessibility-optimized AI-generated (text 3) radiology reports. Ratings were assessed for (A) whether the tone of the report was respectful and took the patient’s situation into account, and (B) whether the report conveyed an empathetic attitude toward the patient’s level of understanding (n=100). Data are presented as box-and-whisker plots, showing medians and IQRs. *P<.05.

Significant differences were observed in patient responses to the tone (Figure 5A; *F*_2,297_=17.72; *P*<.001) and empathy (Figure 5B; *F*_2,295_=49.57; *P*<.001) of the radiology reports across the 3 text groups. Patients rated the tone of the radiology reports as significantly more respectful and considerate of their situation in the simplified text versions (texts 2 and 3) compared with the original AI-generated radiology reports. Similarly, differences were identified in how the reports conveyed an empathetic attitude toward the patients’ level of understanding, with texts 2 and 3 receiving significantly higher ratings than text 1.

#### Guidance and Motivation for Action

**Figure 6:**
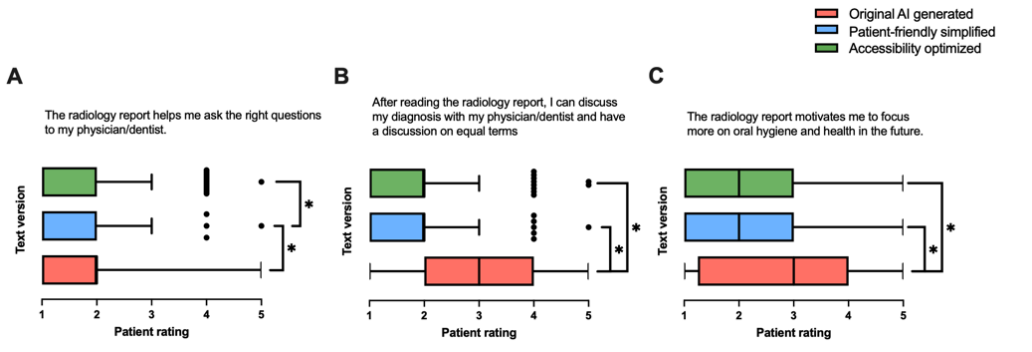
Patient ratings comparing original artificial intelligence (AI)–generated (text 1), patient-friendly simplified AI-generated (text 2), and accessibility-optimized AI-generated (text 3) radiology reports. Ratings were assessed for (A) whether the report helps patients ask the right questions to their physician or dentist, (B) whether the report enables a discussion on equal terms with their physician or dentist, and (C) whether the report motivates patients to focus more on oral hygiene and health in the future (n=100). Data are presented as box-and-whisker plots, showing medians and IQRs. *P<.05.

Patient responses showed significant differences across the 3 text versions regarding the guidance and motivation provided by the radiology reports. Patients were asked if the reports helped them formulate the right questions for their physician or dentist (Figure 6A; *F*_2,295_=9.387; *P*<.001); supported discussions with their health care provider on equal terms (Figure 6B; *F*_2,297_=29.03; *P*<.001); and motivated them to prioritize oral hygiene and health in the future (Figure 6C; *F*_2,297_=8.873; *P*<.001). Median values differed significantly across text groups, with texts 2 and 3 receiving significantly higher ratings than text 1 for facilitating discussions with health care providers (Figure 6B) and motivating patients to focus on oral hygiene and health (Figure 6C).

#### Future Perspective

**Figure 7:**
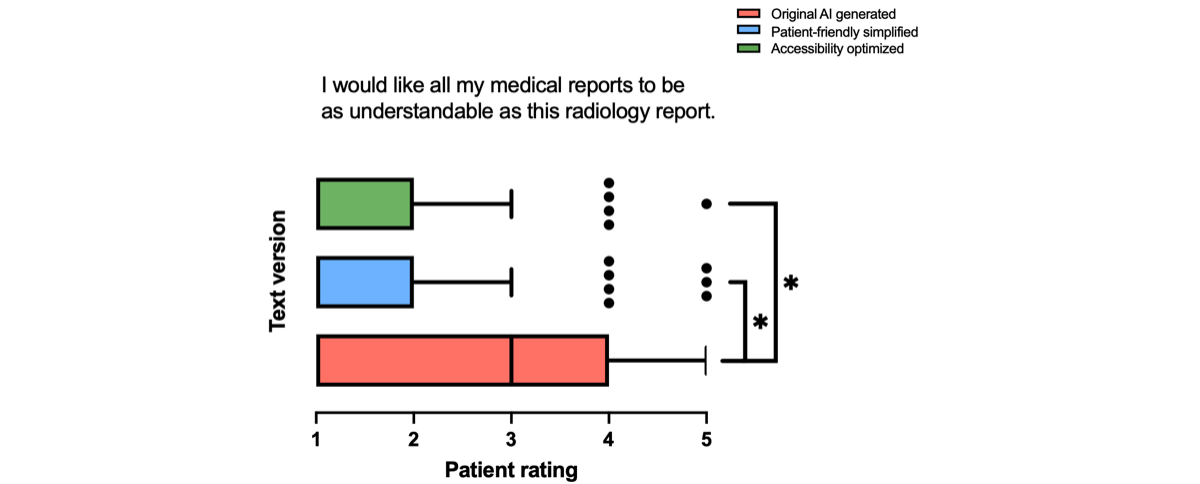
Patient ratings comparing original artificial intelligence (AI)–generated (text 1), patient-friendly simplified AI-generated (text 2), and accessibility-optimized AI-generated (text 3) radiology reports. Ratings reflect the preference for all medical reports to be as understandable as the radiology reports presented (n=100). Data are shown as box-and-whisker plots, displaying medians and IQRs. *P<.05.

Among the 3 text versions, the 1-way ANOVA revealed a significant difference regarding the preference for the understandability of medical reports as presented in Figure 7 (*F*_2,297_=33.75; *P*<.001). Texts 2 and 3 were rated significantly higher than text 1, indicating a strong preference for the simplified versions.

## Discussion

### Overview

Integrating AI into clinical workflows has attracted significant interest due to its potential to enhance efficiency. Therefore, our study aimed to investigate the potential of ChatGPT in improving the clarity and comprehensibility of radiology reports for patients. Simplified versions of AI-generated reports with higher readability scores were consistently rated higher by patients than the original AI-generated reports across all evaluated dimensions, including clarity, structure, tone, and patient engagement. The accessibility-optimized version demonstrated the greatest improvements, indicating that simplification enhances accessibility without compromising the quality of the content. Patients found the simplified reports more empathetic and respectful, which positively influenced their ability to engage in meaningful health care discussions and focus on maintaining health.

### AI in Clinical Documentation and Communication

The use of medical documents must account for the diverse communication needs within health care settings. Health care professionals require detailed and comprehensive reports to ensure accurate clinical decision-making and effective interprofessional communication. In contrast, patients benefit from simplified and accessible documents enabling them to understand their medical conditions and actively participate in treatment discussions [2,3]. In the United States, for example, it is recommended that patient-facing health literature is written at a sixth-grade reading level (age 11-12 years) or lower to accommodate the general population’s literacy levels [29]. Health literacy, defined as an individual’s ability to seek, comprehend, and use health information to make appropriate health-related decisions, is a critical factor in patient communication. Therefore, clinical letters or individual patient reports serve as communication tools to support health literacy and should hence be written in a manner that can be easily understood [30]. However, many patients still receive medical documents intended for professional communication, which are often too complex to comprehend for individuals without medical education. In a recent meta-analysis by Armache et al [31], the readability of 1124 education materials for patients with head and neck cancer was evaluated that was produced by professional societies, hospitals, and other organizations intended to inform patients, promote their engagement, and enhance their adherence to treatment plans. The mean Flesch-Kincaid grade level of these materials ranged from 8.8 to 14.8, with none of them meeting the recommended sixth-grade readability standard. In addition, 8 studies included in the analysis reported inadequate health literacy prevalence ranging from 11.9% to 47.0% [31]. Similarly, another study [32] found that none of the 11 publications by the American Society of Metabolic and Bariatric Surgery met the readability criteria for patient education materials. Similar results were reported for the assessment of online patient education materials for graft-versus-host disease, with an average FRE score of 46.4, underlining the persistent issue of insufficiently accessible medical information [33]. Therefore, these findings highlight the responsibility of health care professionals to diminish preventable literacy barriers by providing patient-centered education materials.

To further address these communication challenges, the integration of AI in health care documentation provides the opportunity to meet the needs of both health care professionals and patients. Although detailed reports support clinical decision-making and interprofessional communication, simplified, patient-friendly texts enable patients to better understand their medical conditions and engage actively in health care decisions. LLMs such as GPT-3.5 and GPT-4 have demonstrated significant utility in summarizing complex data and generating comprehensible information tailored to different audiences [8,34]. This adaptability emphasizes ChatGPT’s potential to enhance communication across various medical contexts, as demonstrated by its ability to provide templates, refine the content of clinical consultation letters, and generate patient letters with a humane tone [35].

Furthermore, ChatGPT has been proven efficient in various methods of documenting standardized patient histories, including typing and dictation, although its readability and accessibility still depend on human refinement [36]. Our findings align with these observations, particularly regarding text structure and readability. Simplified AI-generated texts included more sentences and words than the original AI-generated versions, reflecting efforts to enhance clarity through paraphrasing and additional explanations. Because the study focused on language comprehension in a German-speaking population, translation into English was intentionally avoided to preserve the linguistic features under investigation and prevent bias. As a result, no examples of the texts were included, as the translation would distort the linguistic elements, potentially leading to misinterpreting the nature of the simplification. The process itself involved replacing complex medical terminology with plain language, often accompanied by brief explanations. The tone was adapted to address patients directly in an empathetic and accessible manner. Structural changes were implemented to support readability, with content organized into clearly segmented paragraphs, each focused on a single topic. In the accessibility-optimized and most simplified version, the language approached a conversational style, using short, direct sentences and avoiding subordinate clauses. Medical terms were either explained or excluded. These adjustments are reflected in our findings, as the readability analysis further revealed higher FRE scores, a validated measure for the assessment of text difficulty [22,24,25], for simplified AI texts (56.4 for text 3 and 55.0 for text 2) compared to the original AI-generated text (51.1). While the 2 simplified versions did not differ significantly, the scores for simplified texts approached the range expected for sixth-grade readability (around 60), whereas scores below 50 are typically associated with college-level content.

These results highlight ChatGPT’s ability to effectively simplify medical information and enhance accessibility. Consequently, ChatGPT’s ability to meet the technical precision demanded by health care professionals, as demonstrated in previous studies [21], while simultaneously addressing patients’ need for accessible information, underscores its transformative potential in medical communication.

### Patient Evaluation Versus Professional Evaluation

Most existing LLM outputs prioritize technical quality metrics, such as accuracy and grammar, rather than patient comprehension [34]. Several recent studies have demonstrated the potential of ChatGPT and other LLMs in medical applications but have largely excluded patient input. For example, a recent study [37] reported that chatbot-generated responses to patient questions on social media were rated higher in quality and empathy compared to physician-generated responses. However, this evaluation relied solely on assessments by health care professionals, with no involvement of patients. Similarly, another study assessed the factual correctness and humanness of ChatGPT-generated clinical letters using clinician feedback alone, while another group acknowledged the promise of ChatGPT for providing medical information but emphasized limitations in quality without considering patient perspectives [14,38]. Further studies demonstrated ChatGPT’s capacity to generate understandable and evidence-based responses, such as frequently asked questions about total hip arthroplasty and cirrhosis-related inquiries, but these investigations also relied exclusively on expert grading, thereby limiting their clinical applicability to patient communication [39,40]. In contrast to evaluations conducted by professionals [8], the findings of this study integrate patient feedback through structured questionnaires and revealed that simplified AI-generated reports were consistently rated significantly higher across key dimensions, including clarity, tone, and patient engagement, compared to the original AI-generated version. In selecting a standardized radiology report for this study, a common clinical scenario in which patients receive complex medical documents originally intended for communication between health care professionals was simulated. The use of a consistent, nonpersonalized report reflects the typical patient experience of encountering difficult-to-understand clinical texts. Therefore, the influence of variable clinical content was reduced, while enabling us to investigate the effects of linguistic simplification. Although the original AI-generated report was already comparable in tone and complexity to physician-written documentation, our results demonstrate that targeted simplification significantly enhances patient comprehension. Nevertheless, future research should explore the impact of personalized simplification strategies tailored to individual health literacy levels and clinical contexts.

Notably, these simplified reports significantly enhanced patients’ ability to understand radiological findings, empowering them to engage more effectively in health care discussions. Considering that patients often struggle to interpret medical documents independently and frequently seek clarification from their physicians, this not only increases consultation time but may also contribute to uncertainty, anxiety, or misinformed decisions. Simplifying medical reports can help patients better prepare for clinical conversations, enabling more productive and focused interactions with health care providers. This patient-centered approach not only addresses a key limitation in existing research but also demonstrates the potential of AI-driven solutions to transform medical communication by making complex information accessible and supporting shared decision-making.

### Prompt Design in AI Performance

Previous research validated ChatGPT’s ability to generate high-quality radiology reports from structured checkbox information, producing error-free texts with strong similarity to reference materials and excellent readability using a precise prompt [21]. These findings highlighted the importance of carefully crafted instructions in guiding AI outputs effectively, aligning with the established understanding of prompt design as a pivotal factor influencing AI performance [41,42]. Two distinct prompts were used: the first instructed, “Rewrite the radiology report to make it easier for a patient to understand. Do not leave out any information or content,” while the second specified, “Rewrite the radiology report to make it understandable for patients of all educational backgrounds. Do not leave any information or content.” These prompts were designed to ensure content completeness and accessibility, targeting a broad range of health literacy levels. By adhering to these directives, ChatGPT successfully modified its outputs for patient-facing communication without compromising the integrity of the medical information. This targeted approach underscores the importance of precision in prompt formulation, which directly impacts the relevance and clarity of AI-generated texts [41]. These findings align with previous studies that emphasize the role of precise and detailed prompts in mitigating omissions, inaccuracies, and potential misinformation [42,43]. One study [44], for instance, highlighted those errors of omission accounted for 86% of inaccuracies in AI-generated subjective, objective, assessment, and plan notes, underscoring the necessity of refining input frameworks to enhance data completeness and consistency. While ChatGPT demonstrates strong adherence to clinical guidelines, as seen in findings indicating 97% compliance, its ability to provide tailored medical advice or address nuanced clinical scenarios remains limited [45]. These limitations, coupled with the variability observed in outputs across repeated cases, highlight the importance of ongoing refinement in AI input structures and ethical considerations. By incorporating structured protocols and patient feedback, as demonstrated in this study, AI systems can better address the dual needs of health care professionals and patients, advancing communication and accessibility in clinical settings.

### Limitations and Future Directions

While this study highlights the potential of ChatGPT in generating accessible and comprehensible radiology reports, certain limitations and risks need to be considered. The simplified texts were not systematically evaluated for content completeness, as the study prioritized assessing patient comprehension and feedback over engineering the perfect prompt or achieving absolute precision. Nonetheless, the simplifications successfully produced accessible and understandable radiology reports, as evidenced by the readability indices. Considering the focus on evaluating ChatGPT’s potential for enhancing patient communication, there was no need to further refine or evaluate the content and prompt design. However, future research could explore how to balance simplification with clinical precision to further optimize AI-generated texts. Ethical and legal considerations present critical challenges to the integration of AI-generated content in clinical workflows. Accountability, particularly in cases of errors in AI-generated reports, remains unresolved and requires thorough discussion at both institutional and regulatory levels. These concerns are particularly significant within the sensitive physician-patient relationship, where trust is essential. Overreliance on AI-generated materials for patient communication risks diminishing health care professionals’ direct communication skills, potentially undermining this trust. Consequently, it is crucial to position AI as a complementary tool that supports, rather than replaces, human interaction in clinical practice. The reliability of transferred information remains a key limitation in the use of AI, as health care providers are ultimately responsible for the accuracy of the texts they distribute. Thorough proofreading of AI-generated content is therefore essential. Previous research has highlighted that LLMs such as ChatGPT often face challenges in tailoring outputs to individual patients’ specific needs and circumstances, with nuances in phrasing and contextual accuracy significantly influencing patient understanding and trust [34,46]. Despite these general limitations, this study demonstrated ChatGPT’s strong adherence to instructions, emphasizing the critical role of precise prompt design in mitigating errors. Importantly, no hallucinations [16,17,47] were observed in the AI-generated texts. ChatGPT consistently adhered to its directive to rephrase and restructure existing content without inventing or interpreting additional information, thereby minimizing biases and ensuring reliability. Nonetheless, this study did not systematically evaluate the medical accuracy of the simplified texts. Although efforts were made to preserve the intended meaning during the simplification process, the primary focus of the study was on patient comprehension and readability rather than formal clinical validation. Future research should incorporate expert review to ensure content integrity, particularly when applying simplification to more complex medical information. The generalizability of the findings may be limited by the fact that all participants were recruited from a single center in Germany. Cultural, linguistic, and educational differences can significantly influence how medical information is perceived and understood. To improve external validity, future studies should include more diverse and multicenter patient populations across varying health care settings. These findings underscore the need for collaborative efforts between clinicians, AI developers, and policy makers to establish ethical and legal frameworks that address accountability, accuracy, and trustworthiness in AI-generated content.

In conclusion, this study highlights the significant potential of LLMs such as ChatGPT in generating simplified and patient-friendly radiology reports, demonstrating its ability to enhance patient comprehension and engagement. By integrating patient feedback, the findings emphasize the importance of tailoring AI-generated content to meet diverse communication needs in health care. While ethical, legal, and practical limitations persist, particularly regarding accountability and content reliability, the study underscores the transformative role of AI as a complementary tool in clinical workflows. Future research should focus on refining AI prompts and balancing simplification with clinical precision to further optimize its application in patient-centered care.

## Abbreviations

AI: artificial intelligence
ASL: average sentence length
ASW: average number of syllables per word
FRE: Flesch Reading Ease
LLM: large language model
